# Metal ion removal using waste byssus from aquaculture

**DOI:** 10.1038/s41598-020-79253-7

**Published:** 2020-12-17

**Authors:** Devis Montroni, Giorgia Giusti, Andrea Simoni, Genny Cau, Claudio Ciavatta, Claudio Marzadori, Giuseppe Falini

**Affiliations:** 1 Dipartimento di Chimica “Giacomo Ciamician”, Alma Mater Studiorum, Università di Bologna, via Selmi 2, 40126 Bologna, Italy; 2 DiSTA, Department of Science and Technology of Agriculture and Environment, Alma Mater Studiorum, Università di Bologna, via Fanin 40, 40127 Bologna, Italy

**Keywords:** Pollution remediation, Biomaterials, Sustainability

## Abstract

Byssus is a thread-like seafood waste that has a natural high efficiency in anchoring many metal ions thanks to its richness of diverse functional groups. It also has structural stability in extreme chemical, physical and mechanical conditions. The combination of these properties, absent in other waste materials, has novelty suggested its use as matrix for water remediation. Thus, pristine byssus, upon de-metalation, was studied to remove metal ions from ideal solutions at pH 4 and 7, as model chemical systems of industrial and environmental polluted waters, respectively. The byssus matrix’s uptake of metal ions was determined by ICP-OES and its surface microstructure investigated by SEM. The results showed that the byssus matrix excellently uptakes metal ions slightly reorganizing its surface micro-structure. As example of its efficiency: 50 mg of byssus absorbed 21.7 mg·g^−1^ of Cd^2+^ from a 10 mM solution at pH 7. The adsorption isotherm models of Freundlich and Langmuir were mainly used to describe the system at pH 7 and pH 4, respectively. In conclusion, we showed that the byssus, a waste material that is an environmental issue, has the potential to purify polluted industrial and environmental waters from metal ions.

## Introduction

**Figure 1:**
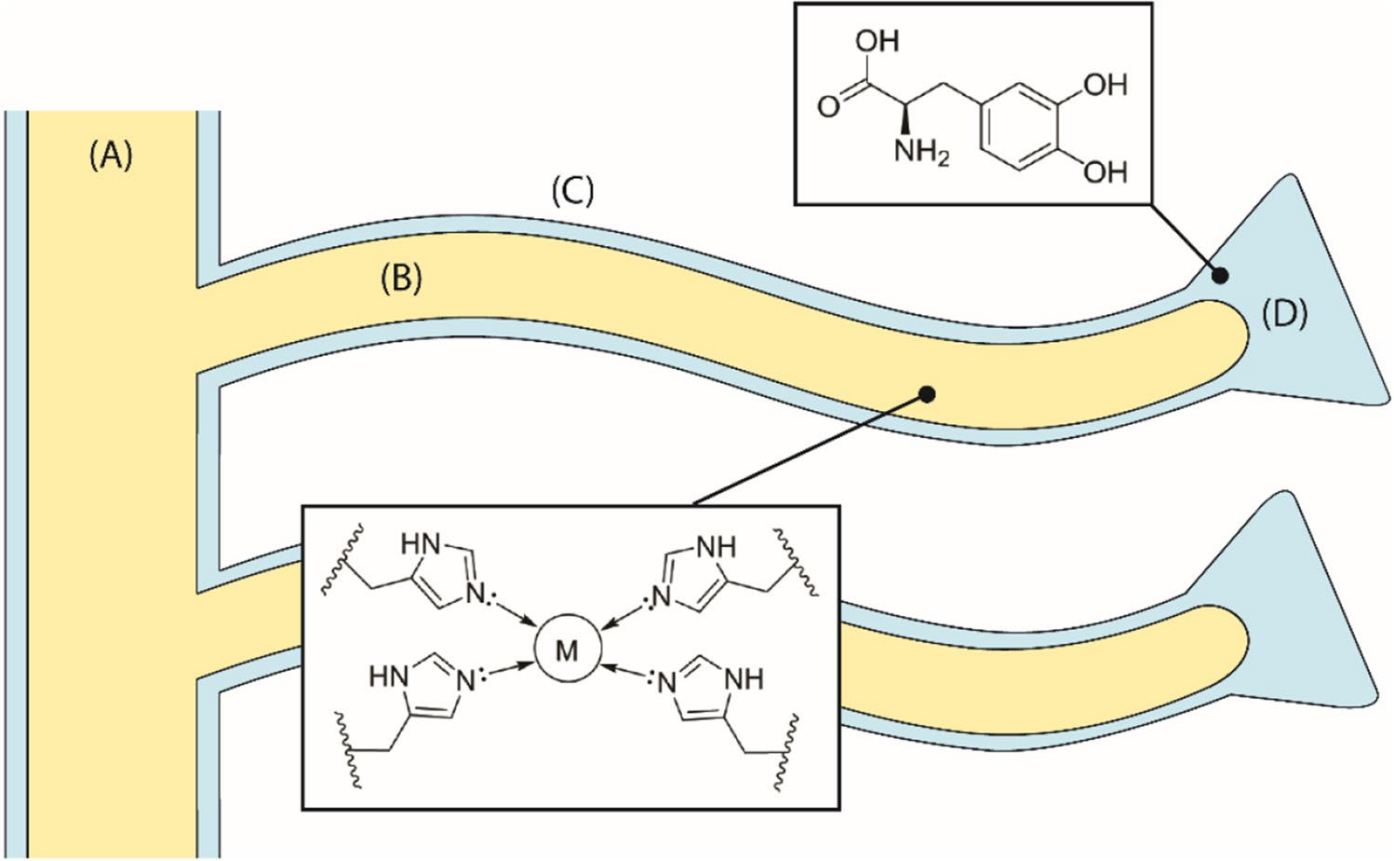
Schematic representation of the byssus. (**A**) The stem, (**B**) the thread core, (**C**) the cuticle, and (**D**) the plaque. In yellow, the collagen- and His-rich regions while, in blue, the DOPA-rich ones are shown.

Metal ions are massively used in many fields, from constructions to medicine, and from electronics to agriculture. As consequence, they are widely dispersed into the environment as pollutants^1,2^. Potential sources of metal ion pollution in aquatic streams are mining refining ores, fertilizer industries, tanneries, battery disposals, paper industries, pesticide utilization, etc.^3,4^. Those activities can introduce in the environment major toxic metal ions like V^3+^, Cr^3+^, Fe^3+^, Co^2+^, Ni^2+^, Cu^2+^, Zn^2+^ Cd^2+^, Hg^2+^, Pb^2+^, etc. that are cause of concern due to their carcinogenicity^5^, bio-accumulation tendency^6^, and persistence in nature^7^. Effluent treatment processes have been designed to avoid metal ions dispersion in wastewater, reducing adverse effects^8^. Some conventional treatments for metal ion removal include complexing, solvent extraction, membrane separation, sedimentation, coagulation, flotation, precipitation, cementation, filtration, electrochemical technique, biological process, reverse osmosis, ion exchange, and adsorption^9^. Unfortunately, each method has its own limitations, such as expensiveness, low efficiency, incomplete removal, sensitive operating conditions, high-energy requirements, and production of waste products or toxic sludge that also require disposal^3^. The adsorption of metal ions using low cost renewable organic materials has gained importance with the time, this because of their high efficiency, easy handling, absence of by-products, high availability, and low cost–effectiveness ratio^10–12^. Among adsorbing matrices good results were obtained using agricultural wastes as cheap disposable adsorbents that also guarantee the reuse of wastes^3,13–18^. A more efficient, but expensive option, was given by chelating polymers as materials developed and synthesized to accomplish this task^19–21^. In the research of low cost metal ion adsorbing materials, the discarded solid residue from the mussel aquaculture (0.5 Mton per year worldwide) should be also considered^22^. Those residues consist of shells, soft tissues, and byssus threads (the latter about 5–15 kton). The waste shells have been used for remediation of anion pollution^23^, as a substrate for heterogeneous catalysts^24^, or as CaCO_3_ source^25^, to cite few examples of many applications. On the contrary, the recovery of byssus has been exploited in few studies^26–29^. The byssus is a protein-based fibrous holdfast that mussels use to anchor to different substrates in sea water and avoid being displaced by waves and currents^30,31^. This material is very rich in binding sites for metal ions due to its unique chemistry and molecular structure. Four regions comprise the byssus ultra-structure organization (Fig. 1), namely: (A) the stem; (B) the thread core; (C) the thread cuticle, and (D) the plaque^32^. As in many natural materials^33^, each different region has a diverse function, associated to a different chemical composition and molecular structure. Dihydroxyphenylalanine (DOPA) residues, which naturally are coordinated to Fe^3+^ in very stable complexes, are localized in plaque and cuticle^34^. The thread core and stem, instead, are mainly composed of collagen-based proteins (called PreCols^35^) having terminal histidine (His)-rich domains able to bind mainly Zn^2+^ and Cu^2+^^36^. Those metal-binding sites have a crucial role in the mechanical and self-healing properties of the byssus. In this natural matrix, His makes up 2 mol.% of PreCols composition while DOPA content reaches 10–15 mol.% in mussel foot protein-1 (mfp-1)^37^. Mfp-1 is the only protein composing the cuticle while one of the five different mfps composing the plaque. Among them, mfp-2 is the most abundant (25 wt.% of plaques) and is characterized by a relatively low percentage of DOPA content, from 3 to 5 mol.%^38^. The remaining 75 wt.% of the plaque is composed of proteins with a higher amount of DOPA residues (e.g. mfp-3 and mfp-5 have DOPA contents higher than 20 mol.%)^39^.

In this research, we aim to exploit the unique composition and structure of the byssus, evolved to bind metal ions, as material for metal ion removal from polluted waters. It has the potential to be a very cheap, high efficient, and disposable or reusable adsorbent. Firstly, the byssus was de-metaled by chelating agents that left its macromolecular composition unaltered^34,40^ and, secondly, tested for metal ion removal application using both metal ions naturally present in the native byssus (Fe^3+^, Zn^2+^, and Cu^2+^) and some metal ions of environmental interest (Al^3+^, Cd^2+^, Co^2+^, Ni^2+^, Mn^2+^, and V^3+^).

## Materials and methods

### Materials

Reagents and solvents were purchased from Sigma Aldrich. They were utilized without any further purification. For each experiment, daily fresh solutions were prepared. A mussel farm close to Fano (Italy) provided the byssus from *Mytilus galloprovincialis*.

### Byssus pre-treatment

The byssus (1–3 wt. %) was collected by hand from the mussels and pre-treated according to the procedure reported in Montroni et al.^29^. Briefly, the collected byssus was washed using tap water and soap until clear washing water. Then the soap was eliminated by rinsing with distilled water and the byssus was stirred twice in ethanol for 30 min and washed again with distilled water for 15 min. The clean and dry byssus was stored in a desiccator under vacuum.

### Byssus de-metalation

The de-metalation process was performed according to the procedure reported by Schmitt et al.^40^, and modified by Montroni et al.^29^. A 0.2 M ethylenediaminetetraacetic acid (EDTA) in a 0.1 M pH 4.3 Tris buffer solution was used as a metal ion chelating agent. The de-metaled byssus was then washed several times in milliQ water to restore the neutral pH and eliminate the EDTA. The air-dried sample was conserved in a plastic Petri dish in a desiccator under vacuum.

### Metal ion removal experiments

Metal ion removal was performed inserting 50 mg of byssus in 2 mL of the buffered solution with different concentrations of metal ions. The experiments were performed in a polystyrene multi-well plate. For each experiment only one metal ion was used. Control experiments were performed to quantify the eventual desorption of metal from the byssus in the buffer solution used. The incubation time was 72 h, afterward, the metal solution was collected. The byssus was then washed twice with 100 µL of the buffer. The latter buffer and the metal ion solution were collected together and diluted to 5 mL using the experimental buffer. The concentration of metal ion in solution was measured using induced coupling plasma optical emission spectroscopy (ICP-OES) in the final 5 mL solutions and in the loading solutions. The detection limits are reported in Table S1. The metal ion loading concentrations used were 0, 0.01, 0.1, 0.5, 1, and 10 mM. Each experiment of incubation was performed in double at 25 °C, two more replicates were prepared and analyzed whether the first results were not in agreement. The buffer used was 50 mM 2,2-bis(hydroxymethyl)-2,2′,2″-nitrilotriethanol (bis–TRIS) for the pH 7 solutions, except for iron (III) solution where 50 mM N,N-bis(2-hydroxyethyl)glycine (bicine) was used. A 50 mM acetate solution was used to obtain the buffer at pH 4.

### Induced coupling plasma-optical emission spectroscopy (ICP-OES) metal measures

All samples obtained from metal ion removal experiments were measured three times, 12 s each, with 60 s of pre-running, using an ICP-OES, Spectro Arcos-Ametek, Inductive Coupled Plasma Optical Emission Spectroscopy with an axial torch and high salinity kit. The Al signal was measured at 117 nm, the Cd at 229 nm, the Co signal at 229 nm, the Cu at 325 nm, the Fe at 232 nm, the Ni at 232.0 nm, the Mn at 258 nm, the V at 293 nm, and the Zn at 214 nm. Certified standards in the experimental buffer were used to prepare the calibrating curves.

### Metal ion quantification within the byssus matrix

The byssus from the metal ion removal experiments was washed three times using 1 mL of Pre-milliQ water to remove experimental buffer traces. The metal inside the byssus was quantified digesting 50 mg of sample. The sample was set in a Teflon holder with 0.5 mL of H_2_O_2_ (30% Carlo Erba, for electronic applications) and 6 mL of nitric acid (65% Honeywell). The holder was set in a microwave oven, Milestone, programmed to operate as follows: 2 min at 250 W, 2 min at 400 W, 1 min at 0 W, and 3 min at 750 W. The digested sample was quantitatively collected and diluted to 10 mL with water, filtered on paper, and analyzed as described in the ICP-OES section. This analytic procedure was verified using a certified reference material (*Lagarosiphon major*, CRM 60; Community Bureau of Reference, Commission of the European Communities). The measure was carried out in duplicate for each sample type.

### Scanning electron microscopy (SEM)

SEM images were collected using a Philips SEM 515 with a tension of 15 kV. The wet samples were glued on carbon tape, dried in a desiccator, and coated with 20 nm of gold prior image them.

## Results

**Figure 2:**
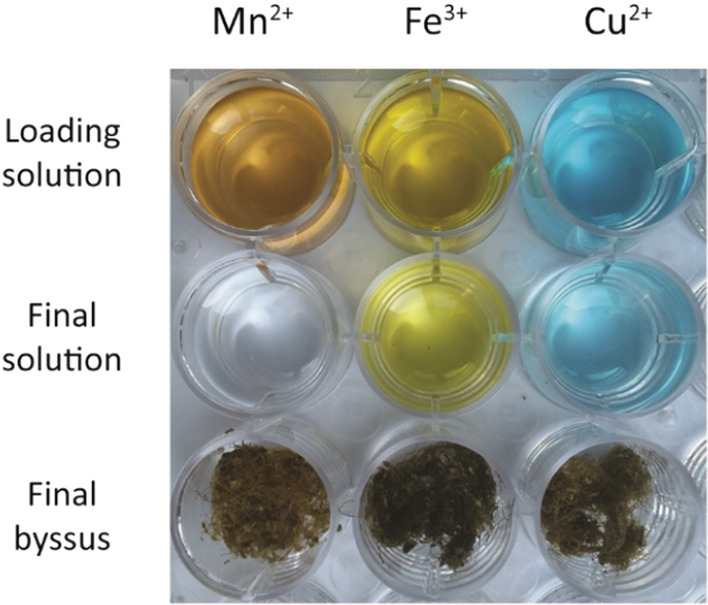
Camera picture of metal solutions and byssus. The 10 mM Mn^2+^, Fe^3+^, or Cu^2+^ solution before and after being in contact with the byssus are reported. After the treatment, a decrease in the color intensity of the solution is observed. The byssus exposed to Fe^3+^ assumed a darker coloration.

The pristine byssus was digested and its metal composition was analyzed (Table S2). The matrix had a 0.5 ± 0.2 wt.% metal content, the major metals were Al, Cu, Fe, and Zn. After the de-metalation process, the metal content of the byssus matrix was 0.122 ± 0.006 wt.% (80 ± 10% less than the pristine), and the main metals were Al and Fe. The concentration of other element did not change considering the standard deviation of the measures. Five different loading concentrations were tested for each metal ion studied: 0.01, 0.1, 0.5, 1, and 10 mM. The amount of adsorbed metal ion was measured as the difference in concentration between the loading and the final solution, after incubating the byssus matrix for 72 h at room temperature (25 °C). The results of the metal ion removal at pH 7 are reported in Table 1 and Figure S1, and the discoloration of three colored metal ion solutions, i.e. Mn^2+^, Fe^3+^, and Cu^2+^, due to the byssus’ metal ion adsorption are shown in Fig. 2. The system was studied in a buffered solution using bis–TRIS, a non-chelating buffer, at pH 7. For Fe^3+^ a bicine buffer was used since iron (III) hydroxide precipitated in bis–TRIS. The analysis of the metal ion solution after the byssus treatment revealed the presence of trace metal ions released from the byssus matrix, most of them were largely below 10 ppb. Only three metal ions were released in an interfering quantity from the matrix, Al (~ 100 µM), Fe (~ 40 µM), and Zn (~ 10 µM). For this reason, reliable data for Al^3+^ and for low loading concentrations of Fe^3+^ and Zn^2+^ were not obtained. It was also difficult to determine the uptake of Mn^2+^ at a concentration lower than 0.1 mM, this was due to the partial overlap of the emission bands of Fe and Mn that made difficult to discriminate the two contributes and detect the small concentration difference between final and loading solution. The byssus matrix was also tested at pH 4 since in this condition His residues are protonated and no more able to chelate metals^41^. Four metal ions were tested at this pH: Ni^2+^ and Cu^2+^ ions that have a high affinity to His, V^3+^ that has a high affinity to DOPA, and Mn^2+^ that has no preferential binding sites. The results of the metal ion removal at pH 4 are reported in Table 2 and Figure S2. Also at pH 4 desorption of metal ions from the byssus matrix was observed, as occurred at pH 7. Al ( ~ 50 µM), Fe (~ 50 µM), and Zn (~ 5 µM) were released from the matrix.

**Table 1:** Metal ion adsorption at pH 7 calculated as the difference between the initial and final concentration of the loading solution.

	0.01 mM	0.1 mM	0.5 mM	1 mM	10 mM
Cd^2+^	0*	(3 ± 1) × 10^–2^	0.4 ± 0.1	0.66 ± 0.02	21.7 ± 0.3
	0*	9 ± 3	18 ± 6	15.7 ± 6	49.6 ± 0.4
Co^2+^	(5.4 ± 0.1) × 10^–3^	(5.0 ± 0.6) × 10^–2^	0.25 ± 0.2	0.43 ± 0.02	9.2 ± 0.1
	26 ± 1	20 ± 2	19 ± 1	16.4 ± 0.7	34 ± 5
Cu^2+^	(6 ± 6) × 10^–3^	(12 ± 2) × 10^–2^	0.63 ± 0.03	0.85 ± 0.07	14 ± 2
	30 ± 30	56 ± 7	52 ± 2	35 ± 3	55 ± 6
Fe^3+^	–	–	–	0.24 ± 0.09	7.5 ± 0.4
	–	–	–	10 ± 4	33 ± 2
Mn^2+^	0*	0*	0.07 ± 0.05	0.47 ± 0.03	16.66 ± 0.01
	0*	0*	6 ± 4	17 ± 1	66.29 ± 0.01
Ni^2+^	(1.3 ± 0.1) × 10^–3^	(6.8 ± 0.5) × 10^–2^	0.399 ± 0.003	0.71 ± 0.09	10.2 ± 0.5
	8 ± 1	29 ± 2	31.6 ± 0.2	29 ± 3	39 ± 2
V^3+^	(2.0 ± 0.2) × 10^–3^	(3.8 ± 0.1) × 10^–2^	0.21 ± 0.02	0.58 ± 0.02	1.3 ± 0.1
	11.4 ± 0.8	21.0 ± 0.6	24 ± 2	26 ± 1	7.5 ± 0.6
Zn^2+^	–	(0.20 ± 0.05) × 10^–2^	0.10 ± 0.01	0.33 ± 0.01	12.8 ± 0.7
	–	1.0 ± 0.3	9 ± 2	14.6 ± 0.6	55 ± 3

For each condition, the results are reported in mg·g^−1^ of the byssus matrix above and the percentage of metal adsorbed from the solution below. The results not reported gave problems due to ion desorption from the matrix. (^*, #^) No difference was observed with the loading solution. The standard deviation is reported.

^#^The desorption concentration of metal ions from the byssus matrix in 2 ml of the buffer-only solutions are reported in table S4.

**Table 2:** Metal adsorption at pH 4 calculated as the difference between the initial and final concentration of the loading solution.

	0.01 mM	0.1 mM	0.5 mM	1 mM	10 mM
Cu^2+^	(2.55 ± 0.03) × 10^–3^	(22.34 ± 0.06) × 10^–2^	1.079 ± 0.002	2.12 ± 0.03	10.4 ± 0.6
	93.6 ± 0.8	93.92 ± 0.06	89.9 ± 0.4	88 ± 1	44 ± 3
Mn^2+^	(2.26 ± 0.08) × 10^–3^	(19.4 ± 0.1) × 10^–2^	0.93 ± 0.02	2.04 ± 0.05	16.96 ± 0.01
	80 ± 3	77.1 ± 0.4	75 ± 1	78 ± 2	66.46 ± 0.01
Ni^2+^	(25.1 ± 0.5) × 10^–3^	(19.7 ± 0.2) × 10^–2^	0.97 ± 0.03	1.92 ± 0.01	4.5 ± 0.2
	89 ± 2	87 ± 1	84 ± 2	82.2 ± 0.2	21.2 ± 0.8
V^3+^	(1.03 ± 0.01) × 10^–3^	(14.6 ± 0.3) × 10^–2^	0.878 ± 0.001	1.76 ± 0.01	15.51 ± 0.07
	82.9 ± 0.3	85 ± 1	96.46 ± 0.03	96.6 ± 0.2	87.8 ± 0.3

For each condition, the results are reported in mg·g^−1^ of the matrix above and the percentage below (^#^). The standard deviation is reported.

^#^The desorption concentration of metal ions from the byssus matrix in 2 ml of the buffer-only solutions are reported in table S4.

The absorption data were interpolated using three different isotherm models: Langmuir, Freundlich, and Dubinin- Radushkevich^42,43^. The statistics of the interpolations are reported in Table 3, Figure S3, and S4. The byssus matrix uptake of different metal ions (reported in mol·g^-1^ from the 10 mM solutions) was compared using a T-test (ν ≥ 2, p = 0.05). This test showed no significant differences among Cd^2+^, Co^2+^, Ni^2+^, Zn^2+^ and Cu^2+^ at pH 7. A significant difference was not detected among Mn^2+^ at pH 4 and 7, and V^3+^ at pH 4. The metal ions absorbed into the byssus were also quantified in few representative matrices. Before the measurements, the byssus matrices were carefully washed with deionized water and digested in acid. The data (Table S3) show that not all the metal ions removed by the solution were in the byssus, suggesting that a fraction desorbed during the washing process.

**Table 3:** Calculated parameters from the fitting of the adsorption isotherms with different models.

	Metal	Langmuir			Freundlich			Dubinin–Radushkevich		
		R^2^	K_L_ (mg^-1^)	q_m_ (mg g^-1^)	R^2^	K_F_ (mg g^-1^)	n	R^2^	K_D_ (mol^2^ kJ^-2^)	q_D_ (mg g^-1^)
pH 7	Cd^2+^	0.915	–	–	**0.978**	0.00107	0.652	0.566	53.3	1.90
	Co^2+^	**0.998**	0.0279	0.494	**0.976**	0.00909	0.938	0.399	0.472	0.485
	Cu^2+^	0.298	0.259	0.0723	**0.912**	0.0267	0.922	0.544	0.304	0.585
	Mn^2+^	0.593	–	–	**0.975**	7.53·10^–6^	0.365	0.805	695	7.13
	Ni^2+^	0.987	–	–	**0.984**	0.00755	0.813	0.552	0.658	0.574
	V^3+^	0.993	–	–	**0.937**	0.00905	1.05	0.766	0.507	0.286
	Zn^2+^	0.925	–	–	**0.993**	6.16·10^–5^	0.450	0.709	32.1	0.895
pH 4	Cu^2+^	**0.989**	0.135	4.43	**0.955**	0.356	1.50	0.756	0.0741	1.99
	Ni^2+^	**0.984**	0.182	1.83	**0.902**	0.224	1.64	0.764	0.0964	1.32
	Mn^2+^	**0.979**	0.0755	2.19	**0.997**	0.144	1.10	0.621	0.164	1.74
	V^3+^	0.613	–	–	0.832	0.622	1.18	**0.918**	0.730	10.6

The R^2^ values from the isotherm model(s) that indicated the best fitting are highlighted in bold.

(–) the values of K_L_ and q_m_ were not reported since negative and with no chemical-physical meaning.

**Figure 3:**
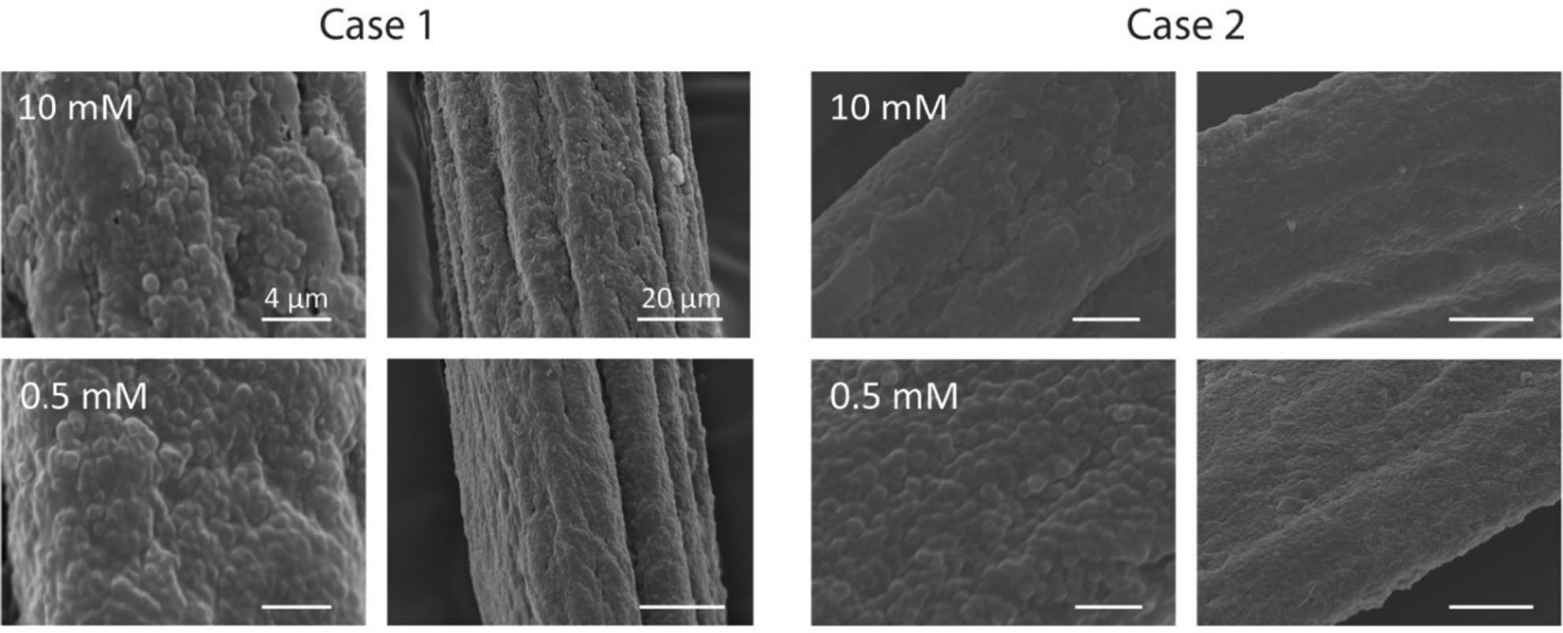
SEM images of the byssus surface. Case 1: Zn^2+^ treated sample at pH 7 where no difference was observed between the two concentrations examined, 10 mM and 0.5 mM. This image is representative of the surface morphology effect of Cd^2+^, Ni^2+^, V^3+^, and Zn^2+^ at pH 7 and for all metals studied at pH 4. Case 2: Mn^2+^ at pH 7 where the 10 mM solution treated sample showed a smoother surface than those treated with the 0.5 mM solution. This image is representative of the surface morphology effect of Co^2+^, Cu^2+^, Fe^3+^, and Mn^2+^ at pH 7. For each condition, two different magnifications are reported.

SEM images were acquired to visualize if the presence of metal ion induces morphological changes on the surface of the byssus matrix. The surfaces of byssus matrices treated with the 10 mM solution and with the 0.5 mM solution were observed. Two cases were discriminated. Case 1: no morphological differences were observed on the surfaces of the samples treated at the two concentrations examined. Case 2: the byssus thread treated using the 10 mM solution showed a smooth surface where globular structures were not visible, differently from threads treated with 0.5 mM metal ion solutions. The metal ions inducing this surface morphology alteration (case 2) at pH 7 were Co^2+^, Cu^2+^, Fe^3+^, and Mn^2+^, while Cd^2+^, Ni^2+^, V^3+^, and Zn^2+^ were in case 1. At pH 4 no metal was able to induce this change (case 1). Representative images of the byssus surface were reported in Fig. 3, while the complete set of images is reported in Figures S5 and S6.

## Discussion

The use of environmentally undesired wastes to purify polluted industrial and environmental waters from metal ions is a field of applied research of growing interest^3,13–15,17^. Thus, in an era environmentally wise, several materials have been tested with a mechanistic approach, even if they do not uptake metal ions in their natural function. The byssus is a complex matrix, which makes difficult to understand the mechanism of molecular uptake of metal ions, evolved to chelate metal ions, a key element in its natural function. The main goal of this research is to test the byssus capability to absorb a wide range of metal ions in two model conditions of pH. To verify this capability, while trying to understand the mechanism of metal ion uptake, simple chemical systems were used. In them only one metal ion was present, allowing us to discriminate the byssus capability uptake, even if this is an important limitation compared to real cases.

Although is reasonable to attribute the metal ions uptake to the high content of DOPA and His residues, our results clearly suggest that many other binding sites are present, which could be no specific. The choice of the metal ions to be used in this research was addressed by a double objective, to prove the capability to uptake toxic and hazardous ions (e.g. Cd^2+^) and to understand the uptake mechanism due to specific interactions with functional groups (e.g. V^3+^ with DOPA^40^). Moreover, to simplify the chemical system, only metal chloride salts dissolved in buffers that avoided the formation of precipitates were used. All the solutions at the start and the end of each adsorption experiment were clear and no insoluble phase was observed. The presence of soluble species was confirmed by a tentative of metal ion speciation performed using the software Visual MINTEQ^44^. It revealed that the metal ions at pH 7 were present as soluble aqueous species, complex ions with chloride^45^, bis–TRIS^46^ and bicine^47^. At pH 4, Cu^2+^, Mn^2+^, and Ni^2+^ were mainly present as aqueous ions. V^3+^ has a complex chemistry^48^ and formed oxyanion complexes at both pH values^49^. However, this ion was still used since it is a proxy for DOPA chelation^40^. Despite the importance of the metal ion speciation, the complexity of the sorption matrix due to its richness of functional groups and different potential adsorption sites makes difficult to understand what metal ion species was adsorbed. This is a weakness from the point of view of understanding the adsorption mechanism, but it represents the strength of this matrix in terms of versatility and efficiency.

The utilization of byssus threads for metal ion uptake required a starting de-metalation process to produce metal ion free binding sites. This process, which reduced of 80 ± 10% of the metal ion content, did not affect the molecular composition, unless at least not in the case of side reactions involving DOPA cross-linking, as already reported^34,40^. After this simple procedure a low amounts of Al^3+^ from the aluminum rich sediments^50^ glued to the plaques, and Fe^3+^ and Zn^2+^ not completely removed from the byssus, desorbed during the uptake experiments, as reported in the result section.

Despite this apparent limitation, the capability of metal ion uptake by the byssus matrix (e.g.: 22 mg·g^−1^ for Cd^2+^, equal to 2 wt.% at pH 7) was higher than many other waste biomasses^3,17^. Similar efficiencies were reported using the waste of jatropha fruit^51^, and the natural biomass collected from an irrigation pond^52^, but these biomasses did not show the versatility of the byssus in terms of pH conditions and metal ion variety.

**Figure 4:**
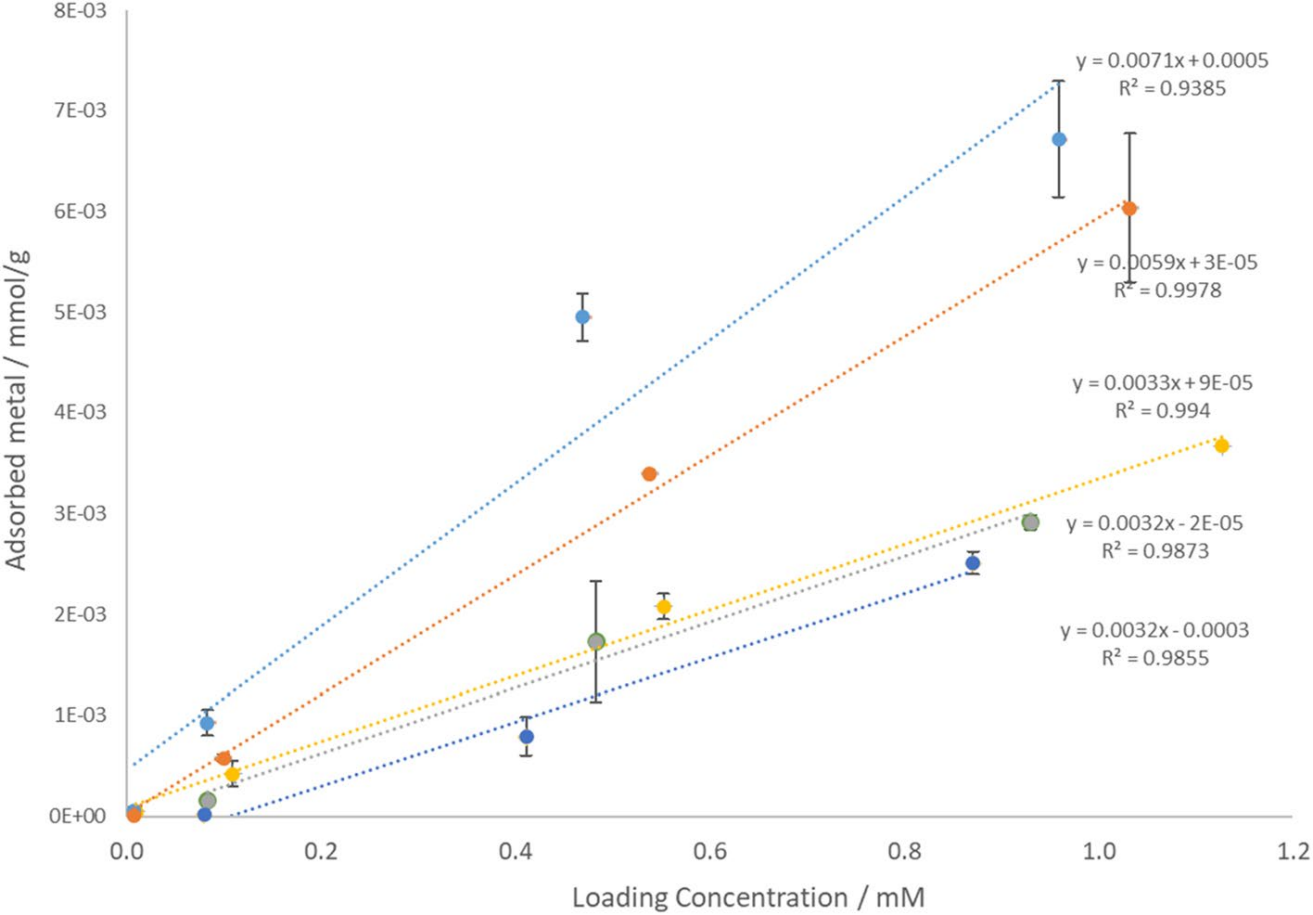
Graph of the adsorbed metal ion (mol·g^−1^) Cd^2+^, Co^2+^, Cu^2+^, Ni^2+^, and Zn^2+^ at the investigated metal ion loading concentrations, excluding the 10 mM solution to avoid artifact due to the saturation of the binding sites. From top to bottom, the elements reported are (light blue) Cu^2+^, (orange) Ni^2+^, (yellow) Co^2+^, (grey) Cd^2+^ and (blue) Zn^2+^. The standard deviation is reported.

In the following discussion, the uptake of the different metal ions at the two pH conditions is analyzed. This discussion is necessary mainly mechanistic, due to the complexity of the matrix that does not allow determining uniquely the chemistry of the binding sites for the different metal ions. Cd^2+^, Co^2+^, and Zn^2+^ were adsorbed by the byssus (as mol·g^−1^) from solution at pH 7 with a similar slope of adsorbed metal ion vs. loading concentration, such slope was dissimilar and higher for Ni^2+^ (Fig. 4). Their adsorption isotherms fitted slightly better a Freundlich isotherm model (Table 3), which describes heterogeneous binding sites, than a Langmuir one, which requires homogeneous mono binding sites. In Freundlich model K_F_ is a constant indicating the adsorption capacity (mg·L^−1^) and the empirical parameter n represents the adsorption energy of the sites^53^. The Langmuir isotherm can be parametrized by dimensionless constant called separation factor (R_L_) that can be calculated by the equation: R_L_ = 1/(1 + K_L_·C_0_), where C_0_ (mg·L^−1^) is the initial metal concentration and K_L_ (L·mg^−1^) is the Langmuir constant. The value of R_L_ indicates if the adsorption is unfavourable (R_L_ > 1), linear and totally reversible (R_L_ = 1), favourable (0 < R_L_ < 1), or irreversible (R_L_ = 0)^54,55^. In the Freundlich isotherm n value for the Cd^2+^, Co^2+^, Ni^2+^, and Zn^2+^ was below 1. This indicated an increasing adsorption with the concentration in solution and was associated with low values of K_F_. The fitting with the Langmiur model produced a realistic value of R_L_ only for Co^2+^, while for Cd^2+^, Ni^2+^, and Zn^2+^ R_L_ indicated an unreal desorption process. We can infer that the complex byssus matrix could have diverse binding sites only for Co^2+^^29,56^, which have to be mono and energetically equivalent at pH 7. The R_L_ value of Co^2+^ was 0.98 and 0.06 in the 0.01 mM and 10 mM solution, respectively, indicating a change of ion adsorption type from linear to irreversible. In addition, the low R_L_ value at high initial Co^2+^ concentrations indicated a favorable adsorption in these conditions, as deducted from the Freundlich isotherm^54^. It was also observed that increasing Co^2+^ concentration the byssus surface morphology changed, suggesting an effect on the byssus surface structure.

At pH 4 His residues were completely protonated and no more able to bind metal ions^41^. In this condition, Cu^2+^ and Ni^2+^ were used as model metal ions with high His affinity and diverse chemistry^57^. V^3+^ was tested as a metal ion representative of those with a high affinity for DOPA residues^40^, and naturally absent in the byssus. Mn^2+^ as metal ion with no specific interaction with both binding sites.

The adsorption data showed that for metal ion concentrations lower than 10 mM the byssus matrix has a higher uptake at pH 4 than pH 7. In those conditions, the lowest percentage of adsorption was 75%, despite the fact that His-based binding sites were protonated. This higher uptake might be due to the speciation of metal ions (i.e. presence of oxyanions) and to the effect of protonation of the physical properties of the byssus matrix. Indeed, collagen-like molecules of the byssus thread can protonate and swell in acid solutions^58^. This physical state should favor metal ion diffusion and made accessible specific binding sites. This agreed with the observation that adsorption isotherms were described better by the Langmuir model than the Freundlich one for Cu^2+^, Ni^2+^, and Mn^2+^. As discussed for Co^2+^, Cu^2+^ and Ni^2+^ showed R_L_ values close to unity in 0.01 mM solutions (0.92 and 0.89, respectively) and close to zero in 10 mM solutions (0.008 and 0.024, respectively). These data indicated a more favorable adsorption for Cu^2+^ than Ni^2+^. Anyway, both the metal ions showed a shift from a linear to an almost irreversible adsorption with an increasing of the initial metal concentration. We could speculate that these metal ions have a stronger binding to the matrix at higher initial concentrations. On the other hand, the Freundlich isotherm fitting gave n > 1 (Table 3), meaning that a lower adsorption was observed by increasing the concentration of the metal solution. This might be explained by a saturation of the binding sites.

When the 10 mM solutions were used, Ni^2+^ and Cu^2+^ reduced their maximum uptake changing pH from 7 to 4 (55 ± 5% and 23 ± 4% less metal respectively). This reduction was significantly similar (T-test, ν ≥ 2, p = 0.05) if calculated as mol·g^-1^ ( ~ 7 × 10^–5^ mol·g^−1^) suggesting that the same number of binding sites were lost, probably His-based.

Cu^2+^, Cd^2+^, Co^2+^, Ni^2+^, and Zn^2+^ showed many similarities, as the same metal adsorption (in mol·g^−1^) at pH 7. Despite that at pH 4 the byssus showed a higher uptake for Cu^2+^ than the Ni^2+^ and that at pH 7 it showed a slope of adsorbed metal ion (mol·g^−1^) vs. loading concentration (mM) (Fig. 4) higher than that of Ni^2+^. These similarities could indicate that Cu^2+^ have access to the same binding sites of Ni^2+^, Cd^2+^, Co^2+^, and Zn^2+^, with a higher affinity or a higher number of non-pH dependent binding sites. The latter could be amino and sulphur ligands or, considering the high amount of Cu^2+^ adsorbed, non-specific binding sites.

As already discussed Cu^2+^, Cd^2+^, Co^2+^, Ni^2+^, and Zn^2+^ adsorption isotherms were described better by a Freundlich model at pH 7 and by a Langmuir model at pH 4. These metals showed a positive correlation between adsorption and initial metal ion concentration at pH 7 and an opposite trend at pH 4 (probably due to the saturation of the binding sites). For Cu^2+^ the latter indication of energetically equivalent mono-binding site (Langmiur model) available at pH 4 was associated to a conservation in the final morphology of the byssus. In fact, Cu^2+^ treated byssus showed a surface morphology related to loading concentration as in case 2 (changed) at pH 7 and as in case 1 (unchanged) at pH 4 (Fig. 3, Figure S5 and S6). This microscopy observation apparently does not fit with the hypothesis of the collagen swelling at pH 4, with the consequent change of morphology upon metal ion uptake. However, it has to be considered that the collagen-like structures of the byssus are located in the bulk of the byssal threads and their swelling should not affect the surface texture and morphology of the byssus.

Ion manganese (II), instead, showed no difference in the uptake using the 10 mM solution in both pHs investigated, relying probably on non-pH-dependent binding sites. Moreover, as observed for other metal ions tested, an increase in the Mn^2+^ uptake percentage at lower concentrations was observed at pH 4. Interestingly, only at pH 7 the 10 mM Mn^2+^ solution induced a change on the morphology of the surface of the matrix. Moreover, at pH 4 the adsorption of Mn^2+^ could be described with both Langmuir and Freundlich isotherm models. This suggested the anchoring on different binding sites, probably made accessible by the collagen swelling, with inversion of concentration dependent behavior as observed for Cu^2+^ and Ni^2+^.

The V^3+^ uptake was higher at pH 4 than pH 7 (16 mg·g^−1^ and 1 mg·g^−1^, respectively) as expected from its speciation. The adsorption isotherms could be described by both Dubinin–Radushkevich model (DR) and Freundlich model. At pH 7, n was close to 1 showing an almost linearity of adsorption with the starting metal concentration. At pH 4, the fitting with the DR model allowed to calculate the average free energy of adsorption [E = (2·K_D_)^−1/2^], 0.828 kJ·mol^−1^^59^. This fitting implies an adsorption mechanism with a Gaussian distribution of energy onto a heterogeneous surface. The higher uptake at pH 4 can be due, among other factors, to a better availability of the DOPA residues. Dopamine, the de-carboxylated form of DOPA, is known to undergo oxidation at pH > 5^60^, preventing the anchoring of metal ions. We suggest that this occurred during the byssus matrix preparation, where the pH is neutral at the end, and the 72 h of incubation at pH 7. That oxidation would explain the relatively low V^3+^ and Fe^3+^ uptake compared to other metal ions. On the contrary, at a pH below 5 dopamine converts to trihydroxylate form, which coordinates metal ions^61^. This change in the redox state of the DOPA can explain why the matrix was able to chelate over 10 times more V^3+^ at pH 4 than pH 7. Differently to the other metals tested, V^3+^ did not fit the Langmuir model at pH 4. Thus, we could assume that this metal ion did not rely on the same non-pH dependent binding sites of the other metal ions tested. The fit of a DR model might be another clue of the DOPA affinity for the vanadium. In fact, the Gaussian distribution of energy on heterogeneous binding sites might arise from the diverse chemical structures that DOPA assume from its oxidation/reduction cycle, when different crosslinking and hydroxylation reactions might take place creating a range of different catechol-based binding sites^59^.

An applicative question could be whether to reuse the byssus after metal adsorption or to dispose it. The byssus can be regenerated by chemical treatment that chelate metals associated to the protonation of its metal ion chelating functional groups. On the other hand, the byssus is a proteic material that can be pyrolyzed, leaving a residue rich of metal ions. Thus, it can represent also a tool to recover metal ions from polluted waters.

## Conclusion

In this study we explored the potential of byssus, a waste material from mussels, to uptake metal ions using simple model chemical systems. This allowed us to evaluate the capability of the byssus matrix to uptake different metal ions in two pH conditions, even if the chemical systems do not represent the complexity of environmental matrices. The byssus is an excellent metal ion-anchoring matrix, especially at pH 4, being suitable and promising for applications, as both disposable and reusable cheap matrix. The results gained in this research, combined with the reported adsorbing capability for aromatic dyes^29,62^, make the byssus a very promising cheap matrix to treat wastewaters from tanneries and paper industries, which usually combine dyes and metal ions to get colored compounds.

## Supplementary Information

Supplementary Information
