# Supplementary material for: Metal ion removal using waste byssus from aquaculture
Source: Sci Rep. 2020 Dec 17;10:22222. doi: 10.1038/s41598-020-79253-7 (PMC7746758; doi:10.1038/s41598-020-79253-7)
Supplement: Supplementary file 1 — Supplementary Information [file 41598_2020_79253_MOESM1_ESM.docx]

Supporting information

# Metal ion removal using waste byssus from aquaculture

Devis Montroni,^1^ Giorgia Giusti,^1^ Andrea Simoni,^2^ Genny Cau,^1^ Claudio Ciavatta,^2^ Claudio Marzadori,^2^ and Giuseppe Falini^*1^

^1^ Dipartimento di Chimica “Giacomo Ciamician”, Alma Mater Studiorum - Università di Bologna, via Selmi 2, 40126 Bologna, Italy.

^2^ DiSTA, Department of Science and Technology of Agriculture and Environment, Alma Mater Studiorum - Università di Bologna, via Fanin 40, 40127 Bologna, Italy.

Table S1: *Detection limits of metal ions*

Table S2: *Metal composition of native and de-metaled byssus reported in µg·g^-1^.*

Table S3: *Metal content in digested matrices.*

Table S4: *Release of metal ions from the byssus matrix into the buffer solutions.*

Fig. S1: *Metal adsorption at pH 7.*

Fig. S2: *Metal adsorption at pH 4.*

Fig. S3: *Fitting of different models at pH 7.*

Fig. S4: *Fitting of different models at pH 4.*

Fig. S5: *SEM of the byssus treated at pH 7.*

Fig. S6: *SEM of the byssus treated at pH 4.*

**Table S1:** Instrumental detection limits (ppb) and wavelength (nm) of analysis of the investigated metal ions in the different matrices used. They were the buffer solution at pH 4 (pH 4), the buffer solution at pH 7 (pH 7) and the buffer digestion (b. d.),

| **metal** | **wavelength** | **pH 7** | **pH 4** | **b. d.** |
| --- | --- | --- | --- | --- |
| Al | 177 | - | - | 13.3 |
| As | 189 | - | - | 6.34 |
| B | 209 | - | - | 3.64 |
| Ba | 455 | - | - | 0.138 |
| Be | 313 | - | - | 0.161 |
| Ca | 184 | - | - | 24 |
| Cd | 229 | 46.1 | 0.469 | 0.203 |
| Co | 229 | 17.2 | 1.01 | 0.17 |
| Cr | 268 | - | - | 0.478 |
| Cu | 325 | 12.3 | 0.738 | 0.056 |
| Fe | 260 | 3.4 | 0.216 | 0.255 |
| K | 767 | - | - | 1 |
| Li | 671 | - | - | 0.086 |
| Mg | 279 | - | - | 7 |
| Mn | 258 | 0.205 | 0.086 | 0.045 |
| Mo | 380 | - | - | 9.54 |
| Na | 330 | - | - | 163 |
| Ni | 232 | 2.76 | 1.1 | 1.24 |
| P | 178 | - | - | 6 |
| Pb | 220 | - | - | 2.27 |
| S | 182 | - | - | 2 |
| Se | 196 | - | - | 11.5 |
| Si | 252 | - | - | 2 |
| Sn | 190 | - | - | 2.82 |
| Sr | 422 | - | - | 0.078 |
| Ti | 191 | - | - | 5.91 |
| V | 293 | 1.73 | 1.43 | 1.14 |
| Zn | 214 | 13.9 | 1.67 | 0.44 |

**Table S2:** Metal content (ppm), of the native byssus and the de-metaled byssus.

| **metal** | **native byssus** | **de-metaled byssus** |
| --- | --- | --- |
| Al | 2300 ± 900 | 1200 ± 900 |
| As | 0.3 ± 0.4 | 0 |
| B | 20 ± 20 | 0 |
| Ba | 30 ± 20 | 8 ± 8 |
| Be | 0.01 ± 0.02 | 0 |
| Ca | 10 ± 8 | 1 ± 1 |
| Cd | 1 ± 1 | 0 |
| Co | 4 ± 3 | 0.5 ± 0.7 |
| Cr | 5 ± 1 | 1 ± 1 |
| Cu | 200 ± 200 | 40 ± 50 |
| Fe | 2000 ± 1000 | 1100 ± 900 |
| K | 1.5 ± 0.6 | 0.9 ± 0.8 |
| Li | 3.8 ± 0.6 | 1.3 ± 0.9 |
| Mg | 0.7 ± 0.3 | 0.16 ± 0.08 |
| Mn | 200 ± 100 | 1.94 ± 0.02 |
| Mo | 20 ± 20 | 30 ± 30 |
| Na | 0.4 ± 0.3 | 0.4 ± 0.5 |
| Ni | 20 ± 20 | 8 ±10 |
| P | 0.3 ± 0.2 | 0.4 ± 0.4 |
| Pb | 0.4 ± 0.6 | 0 |
| S | 3 ± 3 | 6 ± 5 |
| Se | 4 ± 5 | 10 ± 10 |
| Si | 0.05 ± 0.07 | 0.2 ± 0.2 |
| Sn | 0.5 ± 0.5 | 1 ± 1 |
| Sr | 90 ± 70 | 20 ± 20 |
| Ti | 20 ± 10 | 20 ± 10 |
| V | 30 ± 30 | 6 ± 4 |
| Zn | 200 ± 200 | 20 ± 10 |

| **metal** | **matrix** | **solution** | **% retained** |
| --- | --- | --- | --- |
| V | 0.90 ± 0.06 | 1.3 ± 0.1 | 69 |
| Ni | 2.1 ± 0.1 | 10.2 ± 0.5 | 21 |
| Cu | 8.2 ± 0.5 | 14 ± 2 | 58 |

**Table S3**: Analyses of the metal content into the byssus matrices treated using 10 mM solutions at pH 7. The metal content (mg·g^-1^) was measured after digesting the byssus matrices and is reported in the column labelled matrix. The one calculated as the difference of metal ion content in solution before and after the treatment is reported in the column marked solution. The percentage of metal retained in the matrix compared to the one lost from the solution is reported in the last column.

**Table S4:** Concentration of metal ions in 2 ml of buffer-only (0 mM metal ion solution) at pH 4 or pH 7 after 72 hours of exposure to the byssus matrix (see Materials and Methods section). When n.d. is reported the metal ion concentration was below the detection limit of the instrument (Table S1). A low concentration of Cu^2+^, Ni^2+^, and V^3+^ was released at pH 7, while a relevant desorption was observed for Fe^3+^ and Zn^2+^. No metal ion desorption was measured at pH 4.

| **buffer pH 7** | | |
| --- | --- | --- |
|  | **ppb** | **µM** |
| Cd | n.d. | n.d. |
| Co | n.d. | n.d. |
| Cu | 6 ± 1 | 0.25 ± 0.04 |
| Fe | 800 ± 200 | 30 ± 10 |
| Mn | n.d. | n.d. |
| Ni | 8 ± 1 | 0.34 ± 0.04 |
| V | 12.9 ± 0.4 | 0.62 ± 0.02 |
| Zn | 170 ± 40 | 7 ± 1 |

| **Buffer pH 4** | | |
| --- | --- | --- |
|  | **ppb** | **µM** |
| Mn | n.d. | n.d. |
| V | n.d. | n.d. |
| Cu | n.d. | n.d. |
| Ni | n.d. | n.d. |


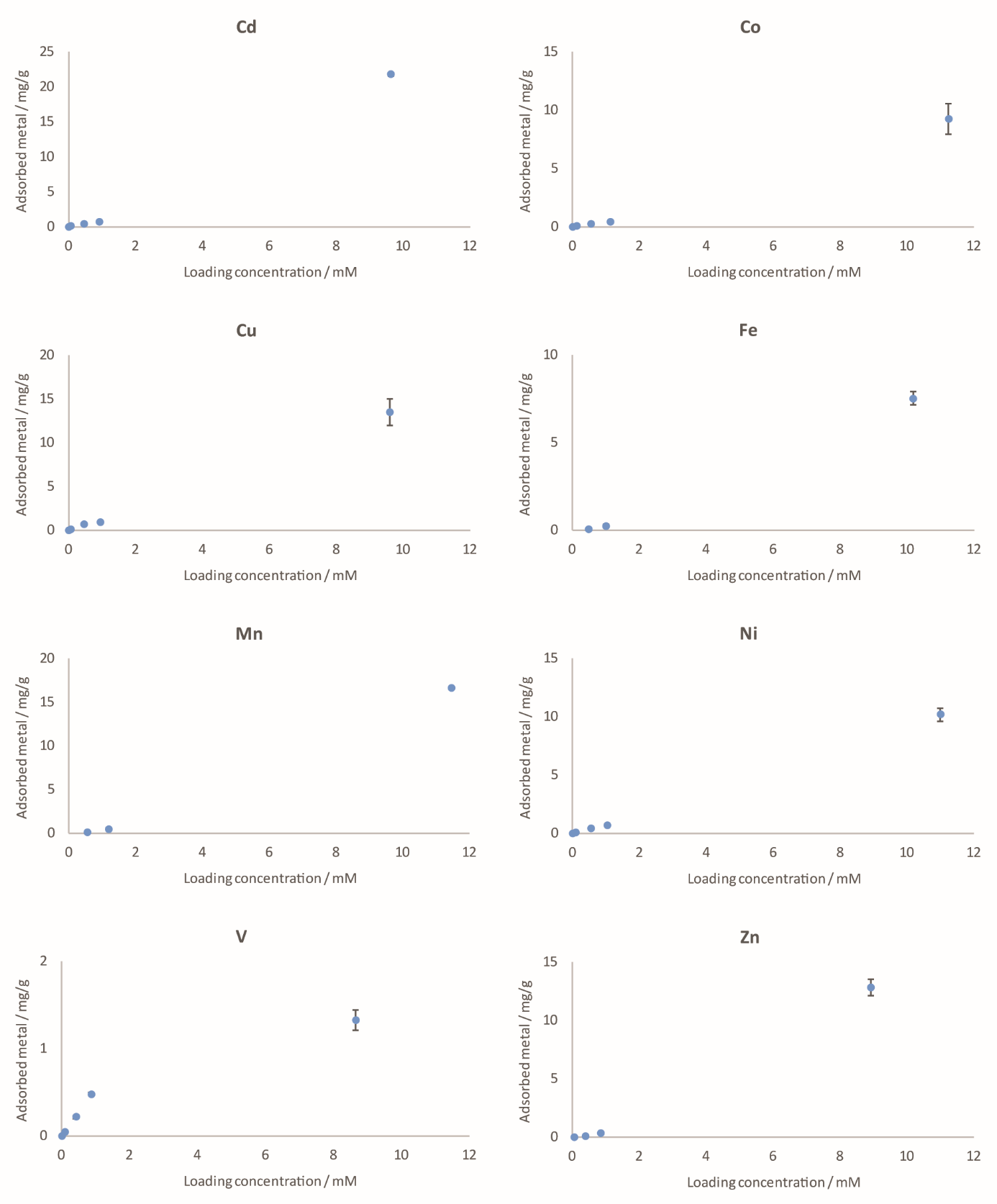


**Figure S1:** Adsorption isotherms of different metal ions by the de-metaled byssus at pH 7.


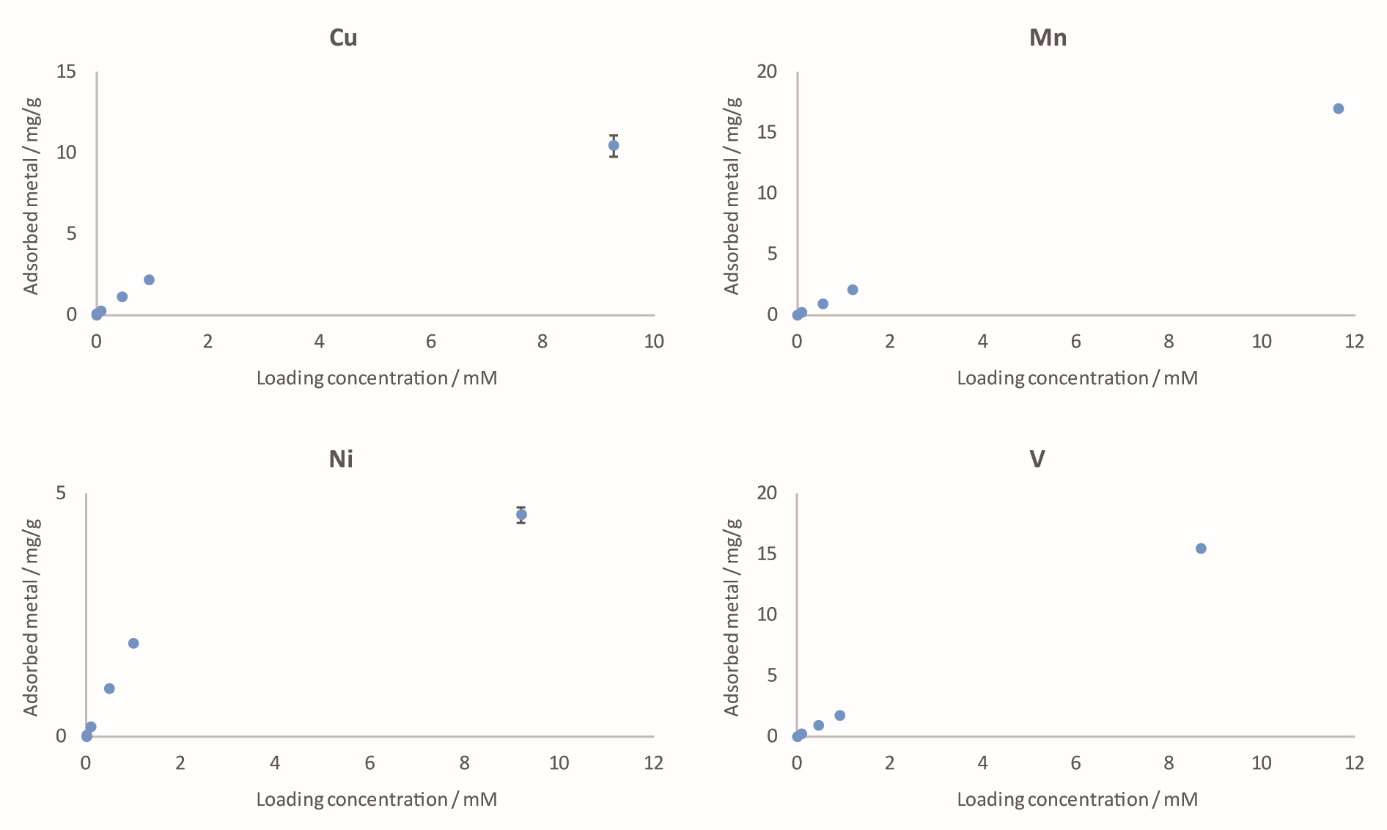


**Figure S2:** Adsorption isotherm of different metal ions by the de-metaled byssus at pH 4.


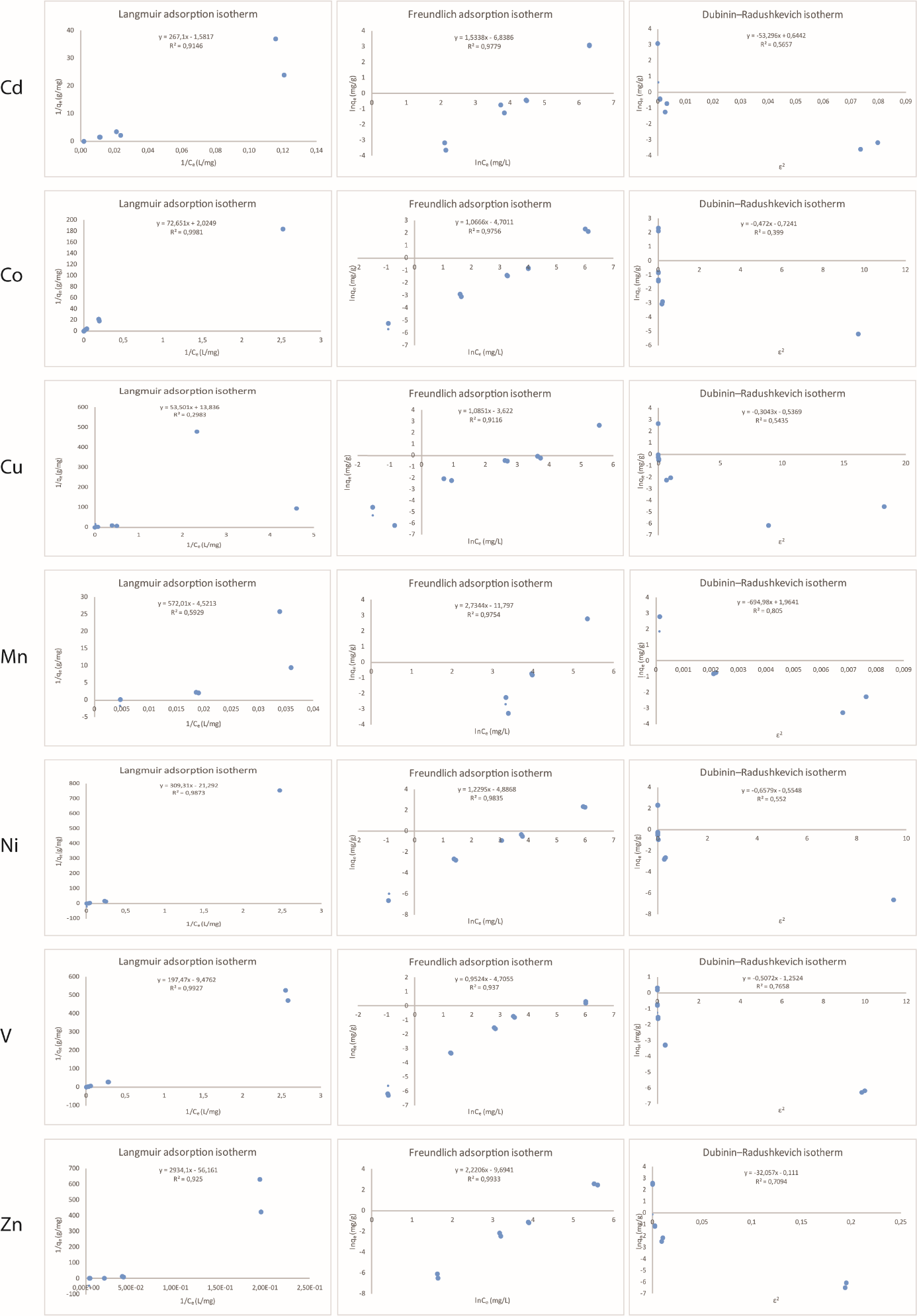


**Figure S3:** Interpolation of different models using the metal ion uptake results at pH 7.


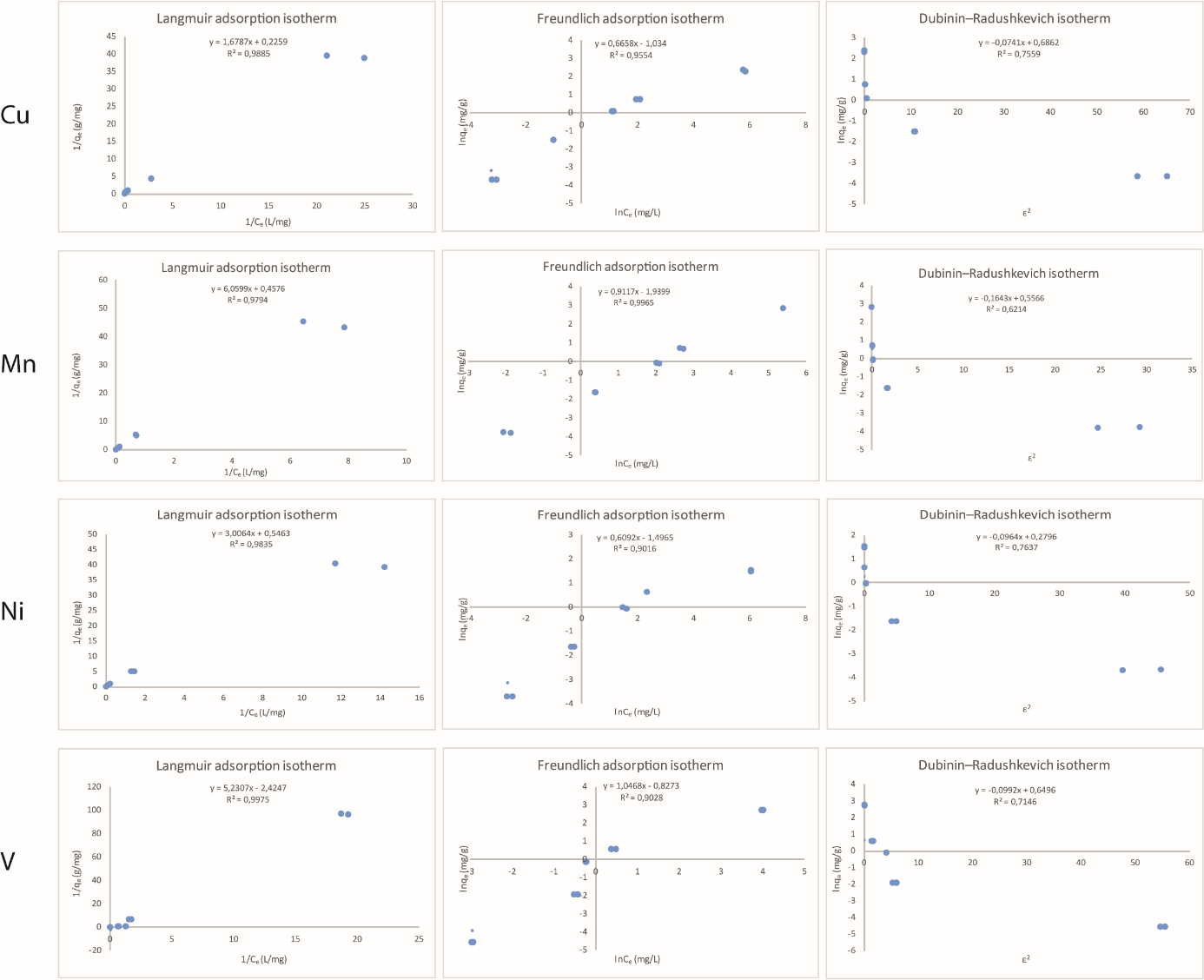


**Figure S4:** Interpolation of different models using the metal ion uptake results at pH 4.


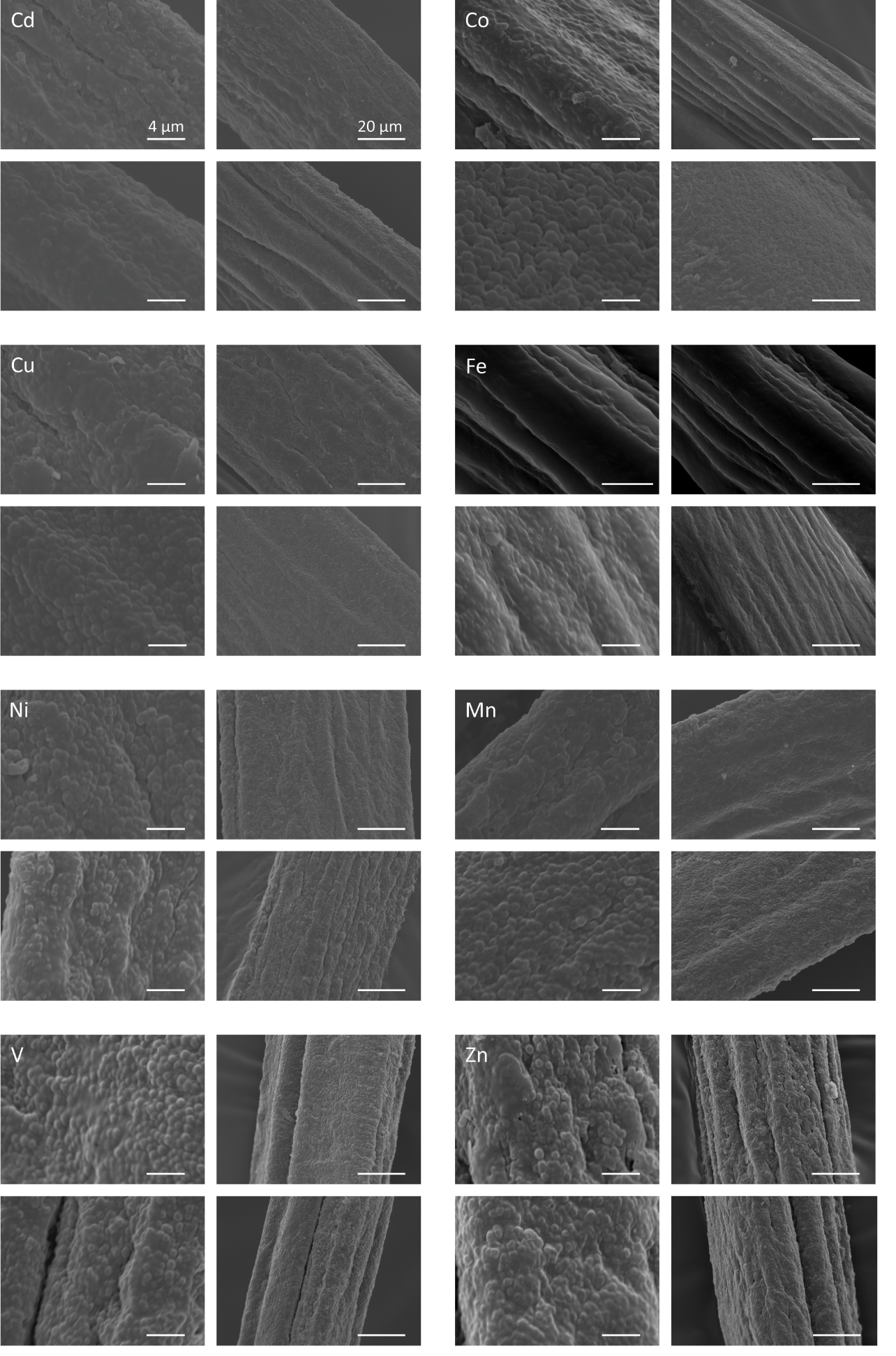


**Figure S5:** SEM images of the byssal thread surfaces treated at pH 7 with different metal ions: Cd^2+^, Co^2+^, Cu^2+^, Fe^3+^, Ni^2+^, Mn^2+^, V^3+^, and Zn^2+^. For each metal the 10 mM solution (above) and the 0.5 mM solution (below) treated byssus matrices are reported with two different magnifications each.


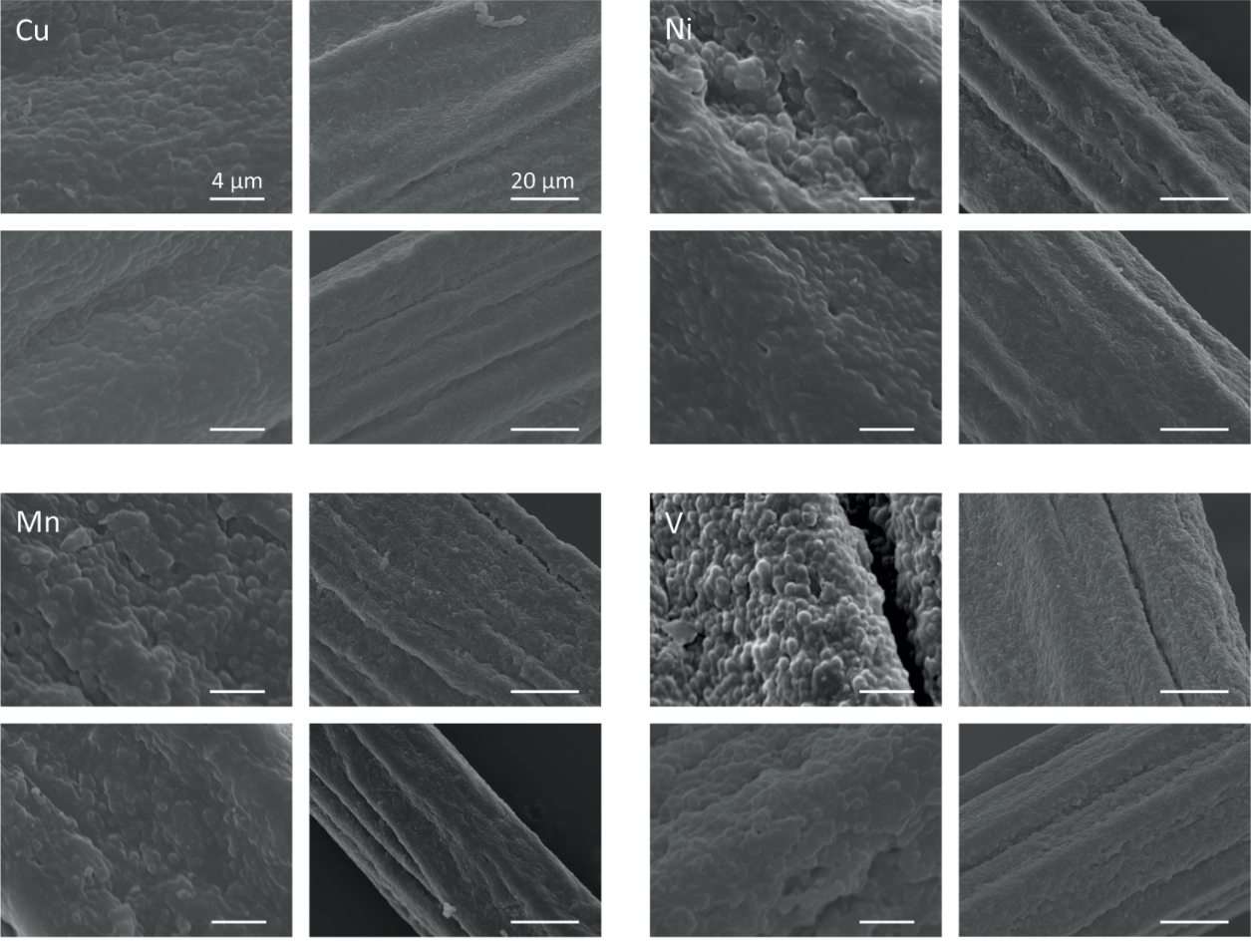


**Figure S6:** SEM images of the byssal thread surfaces treated at pH 4 with different metal ions: Cu^2+^, Ni^2+^, Mn^2+^, and V^3+^. For each metal the 10 mM solution (above) and the 0.5 mM solution (below) treated byssus matrices are reported with two different magnifications each.
